# Nanoparticles in ocular applications and their potential toxicity

**DOI:** 10.3389/fmolb.2022.931759

**Published:** 2022-07-15

**Authors:** Cao Yang, Junling Yang, Ao Lu, Jing Gong, Yuanxing Yang, Xi Lin, Minghui Li, Haiwei Xu

**Affiliations:** ^1^ Southwest Hospital/Southwest Eye Hospital, Third Military Medical University (Army Medical University), Chongqing, China; ^2^ Key Lab of Visual Damage and Regeneration & Restoration of Chongqing, Chongqing, China; ^3^ Key Laboratory of Biorheological Science and Technology, Ministry of Education, College of Bioengineering, Chongqing University, Chongqing, China

**Keywords:** nanoparticles, ocular drug delivery, toxicity, assessment, challenges

## Abstract

Nanotechnology has been developed rapidly in recent decades and widely applied in ocular disease therapy. Nano-drug delivery systems overcome the bottlenecks of current ophthalmic drug delivery and are characterized with strong biocompatibility, stability, efficiency, sustainability, controllability, and few side effects. Nanoparticles have been identified as a promising and generally safe ophthalmic drug-delivery system based on the toxicity assessment in animals. Previous studies have found that common nanoparticles can be toxic to the cornea, conjunctiva, and retina under certain conditions. Because of the species differences between humans and animals, advanced *in vitro* cell culture techniques, such as human organoids, can mimic the human organism to a certain extent, bringing nanoparticle toxicity assessment to a new stage. This review summarizes the advanced application of nanoparticles in ocular drug delivery and the potential toxicity, as well as some of the current challenges and future opportunities in nanotoxicological evaluation.

## 1 Introduction

After decades of development, nanotechnology has been widely used in physics, computer science, biomedicine, and engineering. Among them, biomedicine including nano-delivery systems is an important field of nanotechnology (Bayda, Adeel et al., 2019). Different types of nanoparticles (NPs) have been synthesized for drug delivery due to their more specific targeting, higher stability, better efficacy, and lower side effects compared with conventional drug delivery (Erdoğar, Akkın et al., 2018; Alshawwa, Kassem et al., 2022).

The human eye is a complex organ with intricate anatomical and physiological barriers including the blood-atrial fluid barrier and the blood-retinal barrier, which prevent most of the drugs from reaching the retina, making them much less effective (Zhang J et al., 2021). Recently, the development of nano-delivery systems has been proven to overcome the ocular drug delivery barriers (Peng, Kuang et al., 2022) and improve biocompatibility, stability, efficiency, sustainability, and controllability (Gote, Sikder et al., 2019; Qamar, Qizilbash et al., 2019; Begines, Ortiz et al., 2020; Durgun, Güngör et al., 2020) Nano-drug delivery systems are also considered to be superior to conventional drug therapy in the anterior and posterior segment of the eyes (Yadav, Rajpurohit et al., 2019; Jumelle, Gholizadeh et al., 2020; Kambhampati, Bhutto et al., 2021; Li Q et al., 2021). NPs for drug delivery are mainly classified into organic and inorganic NPs (Zhang J et al., 2021). Organic NPs mainly include polymer NPs, nanomicells, liposomes, quantum dots, nanoemulsion, and hybridized NPs; while inorganic NPs mainly include silica NPs, gold NPs, and carbon nanotubes. In addition, NPs are characterized with strong stability and high light-scattering ability, which can be used in optical coherence tomography (OCT) to improve the early detection and diagnosis of eye diseases (Chen, Si et al., 2021).

Previous studies also showed the application of NPs in ocular diseases, such as cerium oxide NPs for aged-related macular degeneration (AMD), gold NPs for uveitis and AMD, and sliver NPs for fungal keratitis (Pereira, Petronilho et al., 2012; Tisi, Passacantando et al., 2019; Singh R et al., 2020; Shi, Ding et al., 2021). Given NPs possess anti-oxidative stress, anti-inflammatory, anti-angiogenic, and anti-bacterial effects, and their strong permeability and biocompatibility, NPs have been applied in ocular disease treatment (Chan C. M. et al., 2019; Tisi, Passacantando et al., 2019; Zheng, Fang et al., 2019; Gharpure, Akash et al., 2020).

When NPs are used, they tend to be detained in the ocular surface, vitreous cavity, and retinal region, resulting in possible ocular toxicity (Kamaleddin 2017). The toxicity of NPs to humans depends on exposure routes and the features of the NPs (Najahi-Missaoui, Arnold et al., 2020). The eye is a superficial organ and extremely sensitive to external NP toxicity (Zhu, Gong et al., 2019). Moreover, some studies have shown that NPs could enter the body and pass through the blood circulation, causing toxicity in the brain, liver, kidney, and reproductive system (Gupta and Xie 2018). The toxic mechanisms are mainly involved in cellular oxidative stress, mitochondrial damage, chromosome aberration, lipid peroxidation, DNA damage, and cell cycle alteration (Gupta and Xie 2018; Wang R et al., 2018). Furthermore, the size, shape, surface charge, and coating of NPs are closely associated with their toxicity (Kamaleddin 2017; Najahi-Missaoui, Arnold et al., 2020). For example, 15 nm gold NPs exerted more toxicity to liver cells than 60 nm gold NPs (Enea, Pereira et al., 2021). Therefore, it is necessary to clarify the ocular toxicity of NPs which are used in ophthalmology.

## 2 Nanoparticle application in ocular diseases

### 2.1 Carriers for ophthalmic drugs

The eye is divided anatomically into the anterior segment, which contains cornea, iris, conjunctiva, ciliary body, anterior chamber, lens, and aqueous humor; and the posterior segment, which contains sclera, choroid, vitreous humor, and retina (Madni, Rahem et al., 2017). Due to the microenvironment and physiological structures, such as the blood-retinal barrier, blood-aqueous, and corneal barrier, a safe and effective treatment modality has been a matter of inquiry (Awwad, Mohamed Ahmed et al., 2017; Janagam, Wu et al., 2017; Madni, Rahem et al., 2017; Nayak and Misra 2018; Cabrera, Wang et al., 2019). Since invasive treatment modalities such as intravitreal injection and subretinal injection, caused frequent complications include infection, non-invasive treatment has been widely designed (Madni, Rahem et al., 2017; Nayak and Misra 2018; Cabrera, Wang et al., 2019).

**FIGURE 1:**
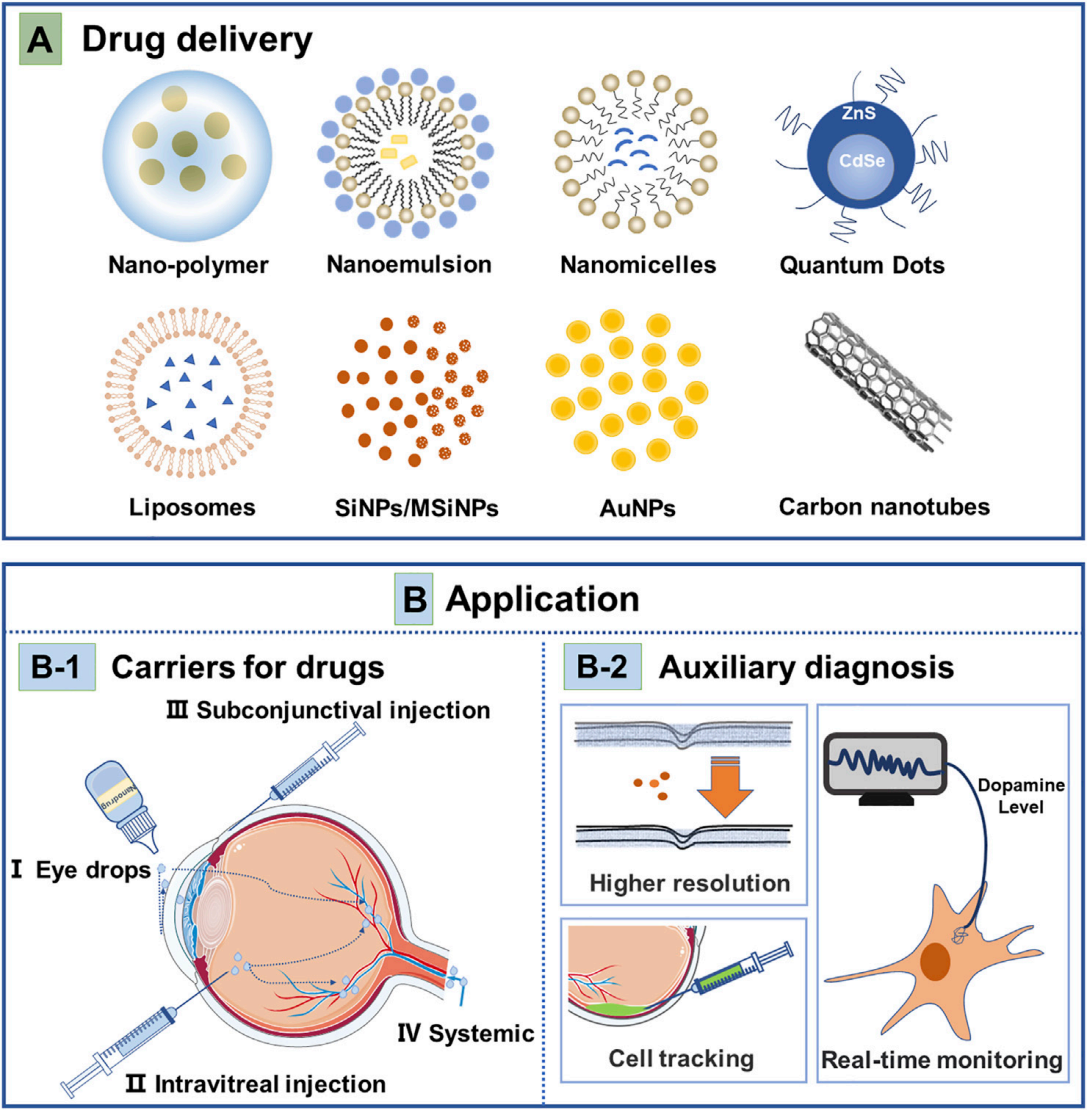
Nanotechnology-based strategies for treatment and diagnosis of ocular disease. **(A)** Drug delivery; The main nanoparticles currently being investigated for drug delivery in ophthalmology are nano-polymer, liposomes, nanoemulsion, nanomicelles, quantum dots, (mesoporous) silica nanoparticles (SiNPs/MSiNPs), gold nanoparticles (AuNPs) and carbon nanotubes. **(B)** Nanoparticles application in ocular diseases. B-1 Carriers for drugs: There are currently three routes of drug delivery by nanocarriers, mainly eye drops, intravitreal injection, subconjunctival injection, and systemic administration. B-2 Auxiliary diagnosis includes nanoparticles as a contrast agent to enhance the resolution of ophthalmic OCT and PAM examinations, nanoparticles fused to cells can tracer transplanted cells in cell transplantation therapy, and nanoparticles allow real-time detection of intracellular dopamine levels.

Topical administration through eye drops cannot easily permeate through the corresponding structures and reach the target tissue (Jumelle, Gholizadeh et al., 2020). The concentration of topical administration is extremely low due to the dilution of tears and drainage from the lacrimal duct, leading to the short duration of action of eye drops. Thus topical administration of ocular therapeutics requires frequent administration to avoid high-concentration drugs caused side effects on local tissues (Diebold and Calonge 2010; Jumelle, Gholizadeh et al., 2020; Schnichels, Hurst et al., 2020). NPs have been designed to increase the bioavailability of the delivered drugs and provided sustained drug release (Diebold and Calonge 2010; Janagam, Wu et al., 2017; Kang-Mieler, Dosmar et al., 2017; Madni, Rahem et al., 2017; Gholizadeh, Wang et al., 2021). Currently, the delivery system of NPs focuses on how to improve the bioavailability, targeting, stability, penetrability, and controllability of drugs (Diebold and Calonge 2010; Madni, Rahem et al., 2017; Srinivasarao, Lohiya et al., 2019). Nanomedicine carriers, such as polymer NPs (Imperiale, Acosta et al., 2018; Begines, Ortiz et al., 2020; Swetledge, Jung et al., 2021), nanomicelles (Grimaudo, Pescina et al., 2019; Durgun, Güngör et al., 2020), liposomes (Khosa, Reddi et al., 2018; Bhattacharjee, Das et al., 2019), quantum dots (Santana, Mansur et al., 2020), nanoemulsion (Singh M et al., 2020), and inorganic NPs (Masse et al., 2019a; Luo, Nguyen et al., 2020) have been developed and widely used in ophthalmic anterior and posterior segment diseases (Figure 1) (Vaneev, Tikhomirova et al., 2021; Wang R et al., 2021)

#### 2.1.1 Organic nanoparticles

##### 2.1.1.1 Polymer nanoparticles

There are two main types of polymer NPs, natural and synthetic ones. Natural polymers include chitosan, hyaluronic acid, sodium alginate, and carboxymethylcellulose sodium; and synthetic polymers include poly(lactic-co-glycolic acid), poly(ε-caprolactone), and poly(ethylene glycol) (Lynch, Kondiah et al., 2019). At present, some medicine for the retina fails to work as expected due to the physical and chemical properties of the drugs and the special anatomical structure of the eye. For example, sirolimus (SIR) is effective for AMD, while SIR cannot be successfully applied to clinical treatment owing to the peculiar environment of the eye and/or physiochemical profile of SIR. Therefore, SIR-loaded poly (lactic-co-glycolic acid) (PLGA) NPs were generated successfully, which were functionalized with chitosan and avoided invasive subretinal or vitreous injection by noninvasive ocular surface drops (Suri, Neupane et al., 2021). It was demonstrated that resveratrol-loaded PLGA NPs with sustained-release efficiency reduced the expression of vascular endothelial growth factor (VEGF) in human retinal pigment epithelial (RPE) cells in the treatment of AMD, avoided frequent invasive intravitreal injection, and improved patient compliance (Bhatt, Fnu et al., 2020).

In neovascular AMD, the conventional approach is vitreous cavity injection of anti-angiogenic factor, while patients will develop drug resistance. The application of trimethoprim for anti-VEGF was limited by intraocular injection, which caused side effects such as increased intraocular pressure and retinal toxicity following local crystallization (Yu, Damico et al., 2006; Sonmez and Ozturk 2012). Hydroxyl-terminated polyamidoamine dendrimer-triamcinolone acetonide conjugate (D-TA) was used in AMD rats (Kambhampati, Bhutto et al., 2021). A single intravenous dose of 3 mg/kg was more effective than a 10-fold dose of free triamcinolone acetonide in preventing choroidal neovascularization (CNV). The NP drug carrier increases the bioavailability of the drug and reduces the side effects.

It was reported that hydroxypropyl methacrylamide-loaded moxifloxacin copolymer NPs were effective in penetrating the cornea in the treatment of bacterial keratitis (Ch, Mishra et al., 2021). Moreover, they allowed for the sustained release of moxifloxacin and increased the retention time in the eye. Furthermore, Mahaling et al. used chitosan-coated poly-L-lactide loaded with azithromycin and tretinoin to create dual-loaded polymer NPs eye drops that reached the retina and choroid and released drugs continuously for more than 300 h, and produced both antibacterial and anti-inflammatory effects (Mahaling, Baruah et al., 2021). The establishment of this dual drug delivery system provides a promising trial for non-invasive drug delivery in ophthalmic drug administration. In addition, Pandit et al. investigated chitosan-coated PLGA NPs loaded with bevacizumab in diabetic retina (DR) model (Pandit, Sultana et al., 2021). The NPs loaded with bevacizumab were injected subconjunctivally into the DR model. It showed that bevacizumab loaded with this NP was more permeable and maintained higher concentrations in the retinal region than bevacizumab alone. Moreover, the nano-drug carrier-loaded bevacizumab lasted for up to 12 weeks, which is longer than the present bevacizumab solution that is approved for clinical use.

##### 2.1.1.2 Nanomicelles

Nanomicelles are a core-shell self-assembled colloidal dispersion with a particle size of 100 nm or less. It is easy to synthesize the desired size and shape of nanomicelles with high stability in an aqueous solution and high cell permeability. Previous studies have shown that nanomicelle formulation improved drug utilization in the eye (Bose, Roy Burman et al., 2021). Bacterial keratitis is a common infection in ophthalmology and easily causes complications, including endophthalmitis and even blindness. The conventional treatment for this type of disease is antibiotic treatment which usually results in antibiotic resistance. Hesperidin (Hes) is a natural compound with strong antibacterial effects, while poor water solubility limits its application (Zhang F et al., 2021). In rabbit models of bacterial keratitis, Hes encapsulated in dipotassium glycyrrhizate (DG-Hes) micelles enhanced the ocular utilization of the drug, improving the antibacterial effects (Zhang J et al., 2021). The successful production of DG-Hes provides a new design for other ocular drugs and improves the bioavailability of existing drugs.

##### 2.1.1.3 Liposomes

Liposomes are spherical vesicles made from biocompatible lipids that resemble cell membranes and have been used for delivering hydrophobic and hydrophilic drugs because of their high stability and permeability (Bhattacharjee, Das et al., 2019; López-Cano, González-Cela-Casamayor et al., 2021). The tear film is the interface between the ocular surface epithelium and the external environment and is divided from the inside out into mucin, intermediate aqueous, and a lipid layer (Pflugfelder and Stern 2020). Dysfunction of the lid gland causes tearing evaporation due to the disruption of the lipid layer of the tear film and usually leads to dry eye, one of the most common diseases of the eye (Chan T. C. Y. et al., 2019). The liposomes are similar to the lipid layer of the tear film and can be applied in the dry eye to restore the normal structure of the tear film (López-Cano, González-Cela-Casamayor et al., 2021). Endophthalmitis is the most serious complication after cataract surgery (Liu, Wilkins et al., 2017). Recently, Wong et al. conducted a clinical trial on liposomes loaded with prednisolone phosphate (Wong, Wong et al., 2022). They injected the NPs in a single subconjunctival injection into patients after cataract surgery. It showed that the NPs were effective in controlling postoperative inflammation. As only five cases were involved in the trial, larger sample size is required to confirm the effect of NPs.

In eye diseases of the posterior segment, ordinary liposomes cannot cross anatomical and physiological barriers. Therefore, liposomes modified by polyhydroxy compounds are characterized with high stability and mobility and can be used to deliver drugs to the posterior segment (Suri, Neupane et al., 2020). Moreover, liposomes encapsulated with ciprofloxacin increased the bioavailability of ciprofloxacin (Feghhi, Sharif Makhmalzadeh et al., 2020), which provides new technical support for the treatment of endophthalmitis.

##### 2.1.1.4 Quantum dots

Quantum dots are nanoscale semiconductor crystals with excellent optical properties (Sarwat, Stapleton et al., 2019). Santana et al. investigated the effect of quantum dot-coupled bevacizumab on retinal neovascularization disease (Santana, Mansur et al., 2020). It showed that the anti-vascular effect of quantum dots coupled with bevacizumab was slightly lower than that of bevacizumab alone due to the coupling rate, and the inflammation in the retina was extinguished 28 days post-treatment. No retinal damage was observed in the quantum dots treated group. However, quantum dots may be deposited in the retinal region, which might produce long-term toxicity. A better characterization of quantum dots and the appropriate dose of the drug is needed to be carried out in future studies.

##### 2.1.1.5 Nanoemulsion

Nanoemulsion (NE) refers to a dispersed liquid with a droplet size of 100 nm or less. NE can be used for ocular drug delivery as it is transparent and has a long shelf life (Dhahir, Al-Nima et al., 2021). It showed that NE equipped with cyclosporine sustainably relieved symptoms of dry eyes (Singh M et al., 2020). It demonstrated that the manufactured NEs could release the drug continuously and the bovine corneal permeability of the two nanoemulsions made could be 2.85 and 2.9 times that of the control group (Mohammadi, Elahimehr et al., 2021). Ismail et al. investigated the feasibility of NEs as a drug delivery vehicle for travoprost in the treatment of glaucoma (Ismail, Nasr et al., 2020). It indicated that the NEs containing travoprost were more bioavailable and successfully prolonged the intraocular pressure (IOP)-lowering effect of the drug compared to conventional Travatan^®^ eye drops. However, the long-term safety of this NE formulation has not been evaluated and the duration of its effects was unclear. These aspects should be studied in depth in the future.

##### 2.1.1.6 Hybridized nanoparticles

Recently, hybridized NPs are regarded as the next generation of nanocarrier development (Mukherjee, Waters et al., 2019; Ferreira Soares, Domingues et al., 2020; Mohanty, Uthaman et al., 2020). For example, lipid polymer hybridized NPs (LPHNPs) are formed using simultaneous self-assembly of polymer NPs and liposomes (Mukherjee, Waters et al., 2019). The core of such hybrid NP is a polymer that can encapsulate drugs, the middle layer is composed of lipids, and the outer layer is encapsulated by lipids and polyethylene glycol. This structure maintains the overall stability during drug delivery, extends drug efficacy, and maintains drug stability by preventing water diffusion through lipids. This may lead to certain developments for systemic ophthalmic drug application. However, more preclinical studies are still needed to confirm their function and biocompatibility. Organic-organic hybrid NPs, inorganic-organic hybrid NPs, and smart conjugated polymers are currently investigated for oral delivery of drugs to provide sustained drug release (Ferreira Soares, Domingues et al., 2020). Smart conjugated NPs, able to respond to pH, temperature, etc., are designed to treat tumors, where the temperature and pH around the tumor will be different from those in the normal tissue. This also provides new sights into the drug delivery for ocular diseases.

#### 2.1.2 Inorganic nanoparticles

##### 2.1.2.1 Silica nanoparticles

Mesoporous silica NPs are usually used as ocular drug delivery vehicles due to their high specific surface area, biocompatibility, and ease of production (Rodrigues, Campos et al., 2020). It was reported that mesoporous silica NPs encapsulated with bevacizumab increased the residence time of the drug in the vitreous cavity and the anti-neovascular efficiency of bevacizumab (Sun, Jiang et al., 2019). Mesoporous silica NPs are hollow and have a high drug loading capacity, which allows them to encapsulate drugs, enhancing their efficacy. It has been reported that mesoporous silica NPs functionalized with 3-aminopropyltriethoxysilane (MSNAPTES) can achieve up to 7% tacrolimus loading (Paiva, Andrade et al., 2021). It showed that vitreous cavity injection of these NPs over 15 days did not cause retinal impairments, including electroretinogram (ERG) changes, neovascularization, and retinal detachment. The delivery of tacrolimus offers a strategy for drug delivery for the treatment of ocular diseases. Wu et al. used low-density lipoprotein (LDL)-coupled mesoporous silica nanoparticles loaded with mitomycin C (MMC) (MMC@MSNs-LDL) for the inhibition of pterygium subconjunctival fibroblasts (Wu, Wang et al., 2020). MMC@MSNs-LDL was found to target the delivery of MMCs to inhibit abnormal proliferation. Moreover, these NPs exerted low toxicity to normal retinal tissues, providing effective drug delivery in ophthalmology. However, the long-term drug release and toxicity were unclear in this study.

##### 2.1.2.2 Gold nanoparticles

Gold NPs are widely used in ophthalmology because of their high stability, biocompatibility, and modifiability (Masse et al., 2019b). Previous studies showed that 20 nm gold NPs could be observed in all layers of the retinal structure after intravenous injection in C57BL/6 mice, indicating that 20 nm gold NPs could cross the blood-retinal barrier (Kim, Kim et al., 2009). This study also showed that gold NPs did not cause damage to the retina. Moreover, recent studies have shown that gold NPs made with functionalized hyaluronic acid could cross anatomical barriers to reach the retinal and choroidal regions (Apaolaza, Busch et al., 2020; Laradji, Karakocak et al., 2021). The gold NPs possess antioxidant and anti-angiogenic properties. By modifying with hyaluronic acid, the gold NPs can cross the physiological barrier of the eye and reach the retina. Once inside the human retina, the hyaluronic acid-gold NPs can deliver relevant drugs to greatly enhance the bioavailability of the drug and its potential therapeutic effects.

##### 2.1.2.3 Carbon nanotubes

Carbon nanotubes (CNTs) are the novel nanocarrier system for the delivery of drugs. CNTs have been widely applied in drug delivery, biosensing, and diagnosis due to their high surface area, stability, and outstanding optical properties (Zare, Ahmadi et al., 2021). The high specific surface area can increase the drug-loading capacity. In addition, Demirci et al. indicated the ability of CNTs to penetrate tumors in an LHT transgenic retinoblastoma mouse model (Demirci, Wang et al., 2020). CNTs can infiltrate throughout the tumor body and act as tumor imaging or drug carriers. However, CNTs also exhibit poor water solubility and dispersion, low biodegradation, and potential toxicity (Negri, Pacheco-Torres et al., 2020).

### 2.2 Ophthalmology assisted diagnosis

#### 2.2.1 Increase the resolution of the image

The diagnostic application of NPs improves the precision of early disease detection in ophthalmology (Scheive, Yazdani et al., 2021). OCT is a common examination to obtain information about the structure of the eye without an exogenous contrast agent. Gold NPs are inert and can permeate through the blood-retinal barrier, suggesting that they are stable and can reach multiple layers of the retina (Chen, Si et al., 2021). Gold NP was used as a contrast agent when performing OCT to improve sensitivity (Figure 1) (Chen, Si et al., 2021) The photoacoustic effect, high scattering, and localized surface plasmon resonance (LSPR) of gold NPs make them backscattering enhancers for OCT and photoacoustic imaging (PAI) (Chen, Si et al., 2021). Nguyen et al. proved that gold NPs could enhance imaging in photoacoustic microscopy (PAM) and OCT (Nguyen, Li et al., 2019). Polyethylene glycol-modified gold NPs (20 nm particle size) were injected into rabbits for PAM and OCT, showing that the signal of PAM was enhanced by 81% and that of OCT by 45%, leading to a clearer visualization of fine vasculature. OCT and PAM are important adjunctive examinations in ophthalmology. The use of NPs enhances the clarity of these ancillary examinations, allowing to detect subtle changes in the retina in the diagnosis and treatment of ophthalmic disease.

#### 2.2.2 Auxiliary cell localization

It was reported that NPs can be modified to target the retina and choroid, improving the diagnostic targeting (Haunberger and Goepferich 2019). Gold NPs were used to label the transplanted cells in the retina to track the grafted cells for at least 1 month with high-resolution images (Figure 1) (Chemla, Betzer et al., 2019). Superparamagnetic iron oxide NPs were used to label photoreceptor precursors in cell transplantation therapy (Ma, Lim et al., 2019). NPs were used to label the corresponding cells giving insights into the location and number of cells. In cell transplantation, this labeling technique may hold the potential for tracking the distribution of cells after transplantation. However, it is important to assess the toxic effects of metal NPs before applying them.

#### 2.2.3 Others

The modification of NPs enables their functions to be expanded. The surface modification of gold NPs with N-butylboronic acid-2-mercaptoethylamine and N-hydroxysuccinimide ester makes it possible to direct detection of dopamine levels in living cells, which will provide insights into the mechanism of myopia and its treatment (Figure 1) (Ren, Zhang et al., 2021). This assay can reveal the body’s condition in real-time at the cellular level and contribute to assessing the effectiveness of treatment. Other modifications of NPs will also enable the detection of many other biomolecules in the eye, which will expand the diagnostic approach to ophthalmology.

## 3 The function of nanoparticles in ocular disease treatment

Previous studies have shown that NPs exert anti-oxidative stress, anti-inflammatory, anti-angiogenic and anti-bacterial effects (Chan C. M. et al., 2019; Tisi, Passacantando et al., 2019; Zheng, Fang et al., 2019; Gharpure, Akash et al., 2020). For instance, cerium oxide NPs are applied for AMD, gold NPs for uveitis and AMD, and sliver NPs for fungal keratitis (Pereira, Petronilho et al., 2012; Tisi, Passacantando et al., 2019; Singh R et al., 2020; Shi, Ding et al., 2021). Next, we present an overview of these NPs according to their function.

### 3.1 Antioxidation (oxidative stress-related eye diseases)

Reactive oxygen species (ROS) is a general term for highly active compounds, including superoxide, hydrogen peroxide, and ozone. When the overproduction of ROS, oxidative stress could cause damage to the body (Ung, Pattamatta et al., 2017). Oxidative stress is an important pathophysiology event in ocular diseases, including the dry eye (Dogru, Kojima et al., 2018), glaucoma (Pinazo-Duran, Shoaie-Nia et al., 2018), diabetic cataract (Obrosova, Chung et al., 2010), AMD (Coleman, Chan et al., 2008; Tisi, Feligioni et al., 2021), and retinitis pigmentosa (Moreno, Mérida et al., 2018). Here, we summarize the recent applications of NPs with anti-oxidative stress effects in these diseases.

Dry eye (DE) is a common ocular surface disease, mainly caused by the instability of tear film and increased oxidative stress injury (Dogru, Kojima et al., 2018). A study on primary mouse corneal and conjunctival cells revealed that glycol chitosan cerium oxide NPs (GCCNP) with a concentration of 1 or 10 μM ameliorated oxidative stress induced by hydrogen peroxide (Yu, Zheng et al., 2019). This improvement mainly resulted from reduced cellular production of ROS, upregulated superoxide dismutase (SOD), and increased mitochondrial membrane potential. In addition, it was found that GCCNP (10 μM) promoted cell migration in the DE model and increased the tear volume after GCCNP treatment. Thus, GCCNP may be a viable treatment for DE. Another study found that grape seed polyphenol NPs (GSP-NPs) in the concentration of 0.5 mg/kg could efficiently reduce the oxidative stress level and the apoptosis of the corneal epithelial cells and conjunctival goblet cells of the DE mice model (Wang T et al., 2021). It suggests that reducing ROS production and oxidative stress levels showed great potential in the prevention or treatment of DE. However, the pathogenesis of DE is complex and also related to immune factors in the body, as well as impaired secretion of the lacrimal gland (Clayton 2018). More effort needs to be put into deciphering the underlying mechanism before clinical application.

Glaucoma is an irreversible blinding eye disease, and oxidative stress is also an important pathogenic mechanism of glaucoma (Pinazo-Duran, Shoaie-Nia et al., 2018). Primary open-angle glaucoma (POAG) is one of the leading causes of irreversible blindness and is related to the decrease of antioxidant enzyme levels in ocular tissues, which is closely associated with the overproduction of ROS (Erdurmuş, Yağcı et al., 2011; Baudouin, Kolko et al., 2021). *In vitro* and *in vivo* studies indicated that the nano eye drops, which were composed of chitosan and ZM241385, were functionalized onto surfaces of hollow ceria NPs (hCeNPs) and loaded with pilocarpine, could open corneal epithelial tight junctions and delivered drug molecules to the ciliary body with the strong anti-oxidant activities and effective in the treatment of glaucoma (Luo, Nguyen et al., 2020). A slow-release nanosuspension SA-9 NP was designed and synthesized, and tested in a dexamethasone-induced ocular hypertensive (OHT) mice model, showing that eye drops of SA-9 NPs significantly lowered IOP (61%) at 3 h post-dose, with the effect lasting up to 72 h (Amankwa, Gondi et al., 2021). It indicates that this novel thiol-containing hybrid antioxidant-nitric oxide donor small molecule is a promising strategy for glaucoma therapy. However, glaucoma is mainly caused by atrial circulation disorders, and the accumulation of large-sized NPs may block the trabecular network, causing atrial circulation disorders and aggravating glaucoma.

Diabetic cataract (DC) is also a common disease resulting from oxidative and nitrosative stress (Obrosova, Chung et al., 2010). CeCl_3_@mSiO_2_ NP eye drops were used in the treatment of DC rat model by streptozotocin intraperitoneal injection (Yang, Gong et al., 2017). The study showed that CeCl_3_@mSiO_2_ NPs increased the level of glutathione, decreased the level of malondialdehyde, and abrogated hyperglycemia-mediated upregulation of advanced glycation end products, lipid peroxidation, and protein carbonylation in the animal lens, delaying the progression of diabetic cataracts. Moreover, the activity of glutathione peroxidase (GPx), superoxide dismutase (SOD), and glucose-6-phosophate dehydrogenase (G6PD) was improved after CeCl_3_@mSiO_2_ NP treatment. CeO_2_-NPs coated with PEG-PLGA (PCNPs) were confirmed to have a stronger antioxidant capacity in lens epithelial cells than CeCl_3_ NPs alone (Zhou, Li et al., 2019). The data showed that 0.5–2 mg/ml PCNPs worked not only effectively inhibited ROS generation in lens epithelial cells, but also acted as glycation inhibitors, which effectively restrained α-crystallin glycation and crosslinking, thereby keeping the lens transparent and alleviating DC. Another human lens epithelial cells *in vitro* study showed that CeO_2_-NPs could simulate the effect of catalase in cells and improve the ratio of glutathione in cells (Hanafy, Cave et al., 2020). Thus, CeO_2_-NPs can protect the lens epithelial cells from oxidative stress caused by hydrogen peroxide, which delayed the development of cataracts (Hanafy, Cave et al., 2021). These data indicated that NPs may be effective in delaying the onset of cataracts. Nevertheless, the etiology of DC is also closely associated with hyperglycemia; thus, blood glucose control should also be considered during the clinical application of these NPs.

AMD is a disease of progressive loss of central vision (Mitchell, Liew et al., 2018). The number of people with AMD is expected to reach 400 million worldwide by 2040 (Mitchell, Liew et al., 2018). The chronic oxidative injury was reported to promote the progress of the AMD (Lem, Davey et al., 2021). Thus, antioxidants are a potential treatment modality for AMD. The CeO_2_-NPs were injected intravitreally into albino rats before being exposed to light damage, showing that CeO_2_-NPs could cross the retina, were detected in the retinal pigment epithelium, and restore retinal function by inhibiting oxidative stress and reducing microglia activation (Tisi, Passacantando et al., 2019). Similarly, Tisi’s group also observed that CeO_2_-NPs were intravitreally injected 3 days before acute light damage (mimic features of AMD), preventing RPE cell death and degeneration in a rat model (Tisi, Flati et al., 2020). Moreover, the epithelial-mesenchymal transition of RPE cells was inhibited by CeO_2_-NPs. Therefore, CeO_2_-NPs might provide a new direction for the clinical treatment of AMD. To date, most of these studies are conducted based on vitreous cavity injections of NPs, which would also be the ones that bring side effects like those of common drug treatments. Future modification of NPs or the use of nano-drug delivery systems will address this issue of the current research.

### 3.2 Anti-inflammation (inflammation-related eye diseases)

The prevalence of chronic inflammatory diseases has gradually increased in recent decades. Non-steroidal anti-inflammatory drugs (NSAIDs) were widely used but exhibited various side effects (Jogpal, Sanduja et al., 2022). Metal and metal oxide NPs, such as gold NPs, silver NPs, and titanium dioxide NPs, exert anti-inflammatory effects (Agarwal, Nakara et al., 2019). Inflammation is an important risk factor for ocular diseases, such as uveitis (Tsirouki, Dastiridou et al., 2018). Gold NPs are topically applied in endotoxin-induced uveitis in rats and can decrease intraocular inflammation by downregulating the TLR4–NF-кB pathway (Pereira, Petronilho et al., 2012). Additionally, curcumin (CUR) was encapsulated in double-headed polyester NPs using gambogic acid (GA)–coupled PLGA. Orally administered PLGA-GA2-CUR in canine models of lens-induced uveitis caused notable aqueous humor CUR levels and was comparable to the clinical effects of anti-inflammatory medications commonly used in the clinic (Ganugula, Arora et al., 2020). The NPs can improve the availability of CUR and reduce the side effects caused by the administration of NSAIDs to the body (Ganugula, Arora et al., 2020). Another *in vitro* study found that hypoxia-induced inflammatory responses in RPE cells could be inhibited by selenium NPs (SeNPs) (Özkaya, Nazıroğlu et al., 2021). The anti-inflammatory mechanism is based on the fact that SeNPs inhibit the TRPM2 pathway and downregulate the expression of TNF-α and IL-1β cytokines. This study indicated that the use of SeNPs is a potential way to prevent or treat retinal inflammation caused by hypoxia. Corneal neovascularization-related diseases are also associated with inflammation. CeO_2_-NPs were used in a rat model of inflammation-associated corneal neovascularization, showing that CeO_2_-NPs decreased the levels of inflammation (Zheng, Fang et al., 2019). Different NPs produce anti-inflammatory effects that might be mediated by different cellular pathways. Thus, understanding the cellular pathways through which NPs act could be helpful for their application. However, most NPs are associated with metals, so the concentration and duration of action need to be considered in further studies.

### 3.3 Anti-angiogenesis (angiogenesis-related eye diseases)

The neovascular AMD, caused by CNV into the photoreceptor layer, can be effectively treated with anti-VEGF (Ambati and Fowler 2012). Current anti-VEGF therapy still facing the challenge of therapeutic instability and potential damage to surrounding tissues (Singh R et al., 2020). Previous studies have shown that gold NPs exhibited anti-angiogenic effects (Mukherjee, Bhattacharya et al., 2005) and could pass through the blood-retinal barrier when injected intravenously (Kim, Kim et al., 2009). Compared with the control group (CNV model mice were injected with PBS), the lesion area of CNV decreased significantly in CNV model mice after gold NP injection (Singh R et al., 2020). Meanwhile, the fluorescein leakage in the lesion area also decreased, demonstrating the antiangiogenic effect of gold NPs. *In vitro* study revealed that the antiangiogenic effect of gold NPs was mediated by inhibiting the Akt/eNOS signaling pathway (Chan C. M. et al., 2019). Proliferative diabetic retinopathy is closely associated with retinal neovascularization (Cheung, Mitchell et al., 2010). A previous study showed that 500 nM silver NPs via subcutaneous injection inhibited retinal vascular regeneration in mice by downregulating the PIK3/Akt pathway (Kalishwaralal, Barathmanikanth et al., 2010).

Zhao et al (Zhao, Gui et al., 2022) found that graphene quantum dots (average particle size of 3.6 nm) inhibited the proliferation and cell migration of human umbilical vein endothelial cells *in vitro*. In the oxygen-induced retinopathy (OIR) model, graphene quantum dots were effective in ameliorating pathological angiogenesis. This antiangiogenic effect was mediated by inhibition of the STAT3-periostin signaling pathway. Graphene quantum dots NPs are a potential treatment for retinal neovascularization diseases. The small particle size of non-metallic NPs may have higher permeability and potentially therapeutic effects compared to metallic NPs. Further comparison of the effects of these emerging graphene quantum dots and metal NPs on retinal neovascular disease is needed. Biocompatibility, therapeutic efficacy, cost of production, and difficulty of fabrication also should be considered to find more suitable therapeutic NPs.

### 3.4 Antibiotic action (microbial-related eye diseases)

The overuse of antibiotics can lead to the emergence of drug-resistant strains, prompting the development of new antibacterial agents. Therefore, the antibacterial activity of some NPs was expected to be used in medicine (Javed, Ain et al., 2022). Metal and metal oxide NPs have been gradually recognized as the next generation of antimicrobial agents (Raghunath and Perumal 2017; Gharpure, Akash et al., 2020). Compared with conventional antibiotics, metal and metal oxide NPs avoid the emergence of drug-resistant strains to a certain extent (Raghunath and Perumal 2017). The antimicrobial effect of NPs was exerted by direct disruption of cytoderm, enzymes, and nucleic acids, rather than the inhibition of cytoderm, RNA polymerase, folate metabolism, and protein synthesis mechanisms of common antibiotics (Raghunath and Perumal 2017).

Sliver microspheres (AgMPs), synthesized using bovine serum albumin and H_2_O_2_, at 16 μg/ml, could effectively combat *Candida* smooth wound infections and were used as an ocular surface drop candidate to treat fungal keratitis (Shi, Ding et al., 2021). AgMPs showed nontoxic effects on corneal epithelial cells when the concentration was below 25 μg/ml. Zinc oxide NPs (ZnO-NPs) are widely used in the biomedical field and have been extensively applied for antibacterial (Singh, Sharma et al., 2021). It was found that the antibacterial activity of ZnO-NPs against *Escherichia coli* and *Staphylococcus aureus* was similar to that of levofloxacin, a commonly used broad-spectrum antibiotic (He, Li et al., 2022). ZnO-NPs are considerable antibacterial agents for contact lenses to reduce the risk of infection from wear (Shayani Rad, Sabeti et al., 2020). It was reported that titanium dioxide NPs catalyzed by UV light can effectively inhibit methicillin-resistant *Staphylococcus aureus* (MRSA) (Javed, Ain et al., 2022). However, the antibacterial activity is based on the photocatalytic activity of the NPs themselves, which may not work in ophthalmology. Moreover, studies have shown that chitosan NPs exhibited excellent drug delivery capabilities and antibacterial capabilities, including resistance to bacteria, fungi, and other food-borne microorganisms (Divya, Smitha et al., 2018; MubarakAli, LewisOscar et al., 2018; Rozman, Tong et al., 2019). Currently, the treatment of fungal keratitis is still a clinical challenge (Sharma, Bagga et al., 2022). The improved penetrability of NPs could be applied to fungal keratitis.

## 4 Toxic effects of nanoparticles on eyes

**FIGURE 2:**
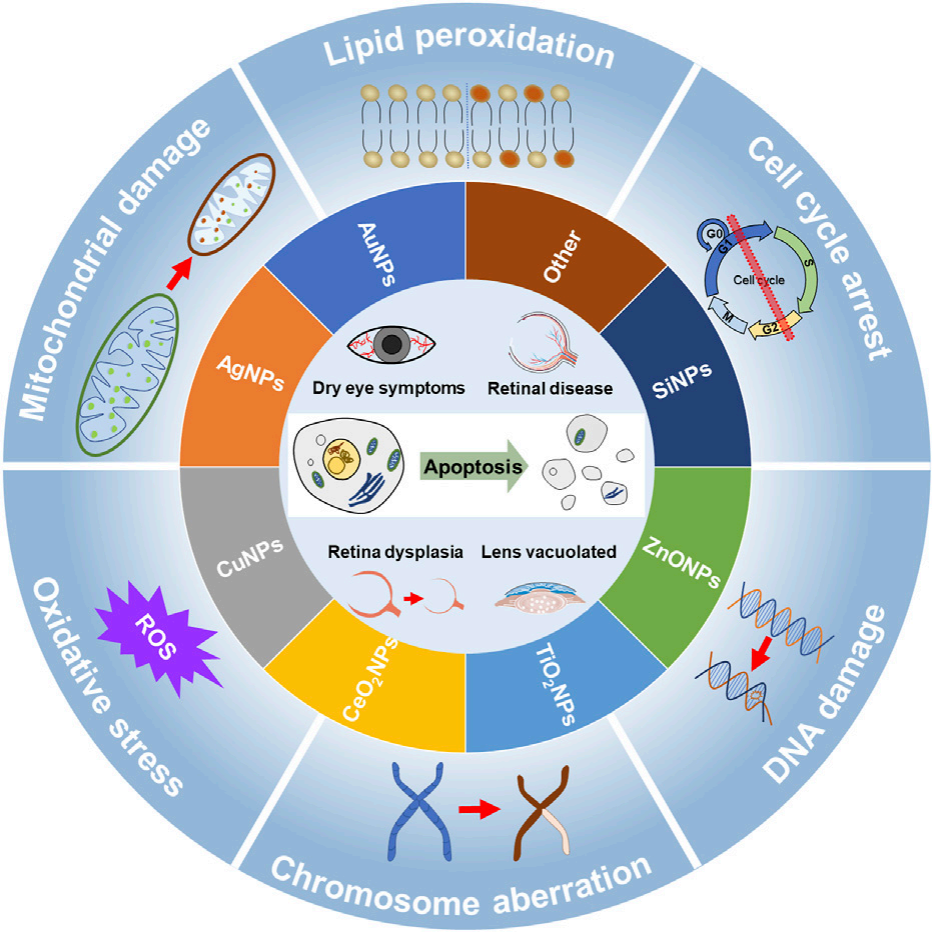
Potential ocular toxicity of nanoparticle and its mechanism. Nanoparticles (inner ring) include gold nanoparticles (AuNPs), silver nanoparticles (AgNPs), copper nanoparticles (CuNPs), cerium oxide nanoparticles (CeO_2_NPs), titanium dioxide nanoparticles (TiO_2_NPs), zinc oxide nanoparticles (ZnONPs), silicon dioxide nanoparticles (SiNPs), and other nanoparticles exert potential ocular toxicity (core). These ocular toxicities are mainly associated with dry eye, retinal disease, abnormal retinal development, and lens vacuolation. The main mechanisms of toxicity (outer ring) include mitochondrial damage, oxidative stress, chromosomal aberration, DNA damage, cell cycle arrest, and lipid peroxidation. These damages eventually lead to apoptosis.

The toxic effects of NPs on major organs, such as the respiratory tract, lung, liver, brain, kidney, skin, and immune system have been investigated in recent years (Ray, Yu et al., 2009; Zhu, Gong et al., 2019). The eye has a certain protective barrier (Zhu, Gong et al., 2019), while NPs with small particle sizes can penetrate the ocular surface barrier and reach the deep part of the eye (Araújo, Garcia et al., 2012). Therefore, NPs could cause cytotoxicity and systemic immune responses at the ocular surface, lens, retina, and even the optic nerve and macula (Figure 2) (Zhu, Gong et al., 2019) In this section, the ocular toxicity of different NPs was reviewed.

### 4.1 Effect of organic nanoparticles on the eye

#### 4.1.1 Polymer nanoparticles

As mentioned above, polymeric NPs are widely used in drug delivery. Therefore, their toxicity assessment is necessary. Chitosan NPs were previously studied for ocular surface toxicity by Enríquez et al. (Enríquez de Salamanca, Diebold et al., 2006). They found that chitosan NPs could be absorbed by the conjunctiva and corneal epithelium within 2 h, but did not cause inflammation of the ocular surface. However, the exposure time in this study was short and the effects of long-term exposure to NPs should be observed. The toxicity of polycaprolactone (PCL), PLGA, and PEGylated PLGA (PEG-PLGA) was studied by Lin et al. (Lin, Yue et al., 2016) using the ARPE-19 cell line and the human retinal vascular endothelial progenitor cell models. It was found that PEG-PLGA did not exert toxicity, while both PCL and PLGA exhibited toxic effects. Further study revealed that PEG-PLGA NPs were detected in the mitochondria, lysosomes, and endoplasmic reticulum after co-culturing for 24 h. The data indicated that lower cytotoxicity of PEG-PLGA was found compared with PLGA. Consistently, Chittasupho et al. evaluated the retinal toxicity of hyaluronic acid-encapsulated PLGA (HA-PLGA) NPs (Chittasupho, Posritong et al., 2018). HA-PLGA NPs exerted nontoxicity to RPE cells. This suggests that the surface modification of the NPs might reduce their toxicity.

#### 4.1.2 Nanomicelles

Previously, the ocular toxicity of methoxy poly(ethylene glycol)-poly(ɛ-caprolactone) (MPEG-PCL) micelles was evaluated by Xu et al. (Xu, Xu et al., 2013). Human corneal epithelial cells, human lens epithelial cells, and RPE cells were exposed to MPEG-PCL. The results showed that the activity of the human corneal epithelial cells, human lens epithelial cells, and RPE cells were not affected when the concentration of the nanomicelles was less than 2 mg/ml. MPEG-PCL was intravitreal injected into rabbit eyes, which did not cause damage to the cornea and retina at a concentration of less than 200 mg/ml. The intraocular distribution of the micelles and their long-term exposure toxicity are needed in further study.

#### 4.1.3 Liposomes

In 1994, Gariano et al. investigated the toxicity of liposomes to the rabbit retina (Gariano, Assil et al., 1994). The retinal examination was conducted after vitreous cavity injection of liposomes by fundoscopy and ERG, showing that liposomes exerted nontoxic effects on the retina. Similarly, the ocular toxicity of liposomes was also evaluated by Abud et al. (Abud, Louzada et al., 2019). Cellular activity and TUNEL assays were performed after ARPE-19 and human retinal progenitor cell exposure to liposomes. The results showed that liposomes did not cause cytotoxicity. Next, liposomes did not cause retinal inflammation or vitreous turbidity after liposome injection. A recent study on the ocular toxicity of liposomes was carried out by Salas et al. (Salas-Ambrosio, Bernad-Bernad et al., 2021). They assessed the subacute toxicity of subconjunctival and vitreous cavity injections of liposomes in New Zealand rabbits, by metabolic, histopathological and biochemical analyses. However, no significant differences were observed compared to the control group. These results suggest that liposomes may be a safe vehicle for ocular drug delivery. However, long-term toxicity assessments are still needed to clarify the potential for clinical applicability.

#### 4.1.4 Quantum dots

Previously, the toxicity assessment of CdSe/ZnS quantum dots in the cornea was studied by Kuo et al. (Kuo, Lee et al., 2011). Quantum dots can penetrate and be retained in the cornea when the epithelial barrier was compromised. The viability of corneal stromal cells was reduced by 50% after being exposed to lower concentrations (5–20 nM) for 24–48 h *in vitro*. However, quantum dots could remain in the corneas of mice for up to 26 days. The long residence time of heavy metals may cause damage to the cornea. Therefore, long-term toxicity assessments are needed in further study. The safety of CdSe quantum dots was also investigated by Jackson et al. (Jackson, Mandava et al., 2021). The results showed that quantum dots can maintain and improve visual acuity in patients with retinitis pigmentosa. However, quantum dots did not cause damage to the retina. This may be caused by the surface modification accomplished by coating ZnS onto the CdSe quantum dots.

#### 4.1.5 Nanoemulsion

Recently, Samimi et al. evaluated the ocular toxicology of fluconazole NE *in situ* gel formulations (Samimi, Mahboobian et al., 2021). The data showed that unloaded NE formulations at concentrations greater than 1% caused a 26% decrease in cell viability when RPE cells were co-cultured for 24 h. Nontoxic effect on cell viability was found at concentrations of 0.1 and 0.5%. The hen’s egg test-chorioallantoic membrane (HET-CAM) test was also carried out, showing that the fluconazole NE formulation did not cause an irritation response. The Draize irritation test on rabbits’ eyes showed the score was zero, while the conjunctival redness in one rabbit’s eye gradually disappeared after 1 h of eye drops. However, the long-term safety of NEs was not evaluated in this study.

### 4.2 Effect of inorganic nanoparticles on the eye

#### 4.2.1 Gold nanoparticles

Gold NPs can be used as diagnostic and therapeutic tools for vision defects (Masse et al., 2019a). Söderstjerna et al. evaluated the toxicity of gold NPs on the mouse retina (Söderstjerna, Bauer et al., 2014). In this study, 20 and 80 nm gold NPs caused oxidative stress and apoptotic damage to retinal cells at low concentrations (0.4 μg/ml 80 nm NPs and 0.0065 µg/ml 20 nm NPs). In addition, obvious neurotoxicity including the toxicity to photoreceptors and the activation of glial cells was observed, which will lead to visual impairment or even blindness. Moreover, toxic effects of NPs on the embryonic development of zebrafish were also observed (Kim, Zaikova et al., 2013). N, N, N-trimethylammoniumethanethiol-functionalized gold NPs caused abnormal eye development as well as hyperpigmentation in zebrafish. *In vitro* study of RPE cells, the toxicity of gold NPs was closely related to their shape, size, and concentration as well as surface area (Karakoçak, Raliya et al., 2016). On the contrary, the histological and electrophysiological assessment of the retina revealed nontoxic effects on retinal integrity and function after intravitreal injections of 160-nm-sized gold nanodisks at a concentration of 10 p.m. in mice (Song, Wi et al., 2017). Earlier studies also found nontoxic effects on the retina and optic nerve in rabbits within 1 month of intravitreal injection of gold NPs at a concentration of 67 and 670 μm/0.1 ml (Bakri, Pulido et al., 2008). This inconsistency may be caused by 1) the different exposed models used; 2) the functionalization of gold NPs and the positive charge on the surface; 3) the particle size, shape, and concentration of the NPs. These data indicated that the characteristic of the NPs plays an important role in the toxicity of the NPs.

#### 4.2.2 Silver nanoparticles

Silver NPs have been widely used for anti-inflammatory, antibacterial, antiviral, and anticancer action. However, silver NPs also exhibit genotoxicity, neurotoxicity, and cytotoxicity (Choudhary, Singh et al., 2022). It was reported that exposure to 16.7 nm silver NPs at 100 μg/ml produced toxic effects on the corneal cells of rabbits (Lee and Park 2019). Exposure to 0.4 mg/L silver NPs did not affect the retinal development of zebrafish embryos, but caused abnormalities in the development of the lens as illustrated by visible vacuoles, and down-regulated expression of some lens genes and protein (Zhang et al., 2018b). Previous studies have demonstrated that silver NPs caused damage to humans as illustrated by DNA damage, chromosome aberration, and cell cycle arrest even at low concentrations (AshaRani et al., 2009). Moreover, silver NPs exerted toxic effects on ARPE-19 cells in a dose-dependent manner after exposure to 0.2, 1, 5, 25, and 125 μg/ml silver NPs solution for 24 h. The cytotoxicity was mainly manifested by the overproduction of ROS, increased G1 phase cells, and mitochondrial apoptosis (Quan, Gao et al., 2020). In our surrounding environment, ionic silver will be released from products and can be adsorbed on mesoporous silica NPs (Chen, Zhu et al., 2020). In our previous study, the toxicity of silver-loaded mesoporous silica NPs on human corneal epithelial cells was evaluated and found that mesoporous silica NPs not only mediated alterations in the MAPK pathway, but also altered the mRNA surveillance signaling pathway leading to genotoxicity (Chen, Zhu et al., 2020).

#### 4.2.3 Copper nanoparticles

Copper NPs (CuNPs) have been used in chemical synthesis, biotechnology, antimicrobial agents, agricultural production, cancer treatment and diagnosis (Ameh and Sayes 2019; Pohanka 2019; Naikoo, Al-Mashali et al., 2021). However, the toxicity of CuNPs should be taken into account. As previous studies have shown, intraperitoneal administration of 3–13 mg/kg of CuNPs to female rats for 14 days revealed a reduction in uterine weight and activation of Caspases 3, 8, and 9, as well as BCL-2 and Bax, resulting in oxidative stress and apoptotic events (Pohanka 2019). During pregnancy, NPs can easily cross the placental barrier and impact fetal retinal development (Dugershaw, Aengenheister et al., 2020; Wang and Wang 2020). The study found that CuNPs, at concentrations of 0.15 mg/L, 0.25 mg/L, 0.5 mg/L, and 1 mg/L, can lead to abnormal eye development of zebrafish embryos in a dose-dependent manner (Zhang et al., 2018a). It was further found that the developmental defects in the zebrafish embryonic retina from CuNPs exposure were caused by the upregulation of endoplasmic reticulum stress and ROS, mediating embryonic cell apoptosis (Zhao, Sun et al., 2020).

#### 4.2.4 Cerium oxide nanoparticles

CeO_2_-NPs, as described above, have excellent antioxidant stress effects and have been shown to reduce photoinduced retinal degeneration. However, a recent study has shown that CeO_2_-NPs exerted toxic effects on human RPE cells (Ma, Li et al., 2021). The study showed that 15 nm CeO_2_-NPs at the concentration of 25 μg/ml reduced the viability of ARPE-19 cells, induced excessive ROS production, and caused mitochondrial dysfunction (Ma, Li et al., 2021). In addition, the toxicity of CeO_2_-NPs coated with modified ethylene glycol (EGCNPs) on human lens epithelial cells also was evaluated by Hanafy et al. (Hanafy, Cave et al., 2020). The data showed that EGCNPs concentrations of 200 μg/ml did not cause toxicity to human lens epithelial cells. However, concentrations of greater than 400 μg/ml induced excessive production of ROS, which mediated mitochondrial damage and triggered apoptosis. However, previously, Pierscionek et al. found that 5 and 10 μg/ml of CeO_2_-NPs did not cause significant toxicity to the human lens epithelial cells (Pierscionek, Li et al., 2010). Their subsequent study also found that CeO_2_-NPs with a particle size of 5.4 nm at concentrations of below 100 μg/ml did not cause toxicity to human lens epithelial cells when exposed for 48 h (Pierscionek, Li et al., 2012). The differences could be caused by the exposure time and levels and the particle size of CeO_2_-NPs.

#### 4.2.5 Titanium oxide nanoparticles

Titanium dioxide NPs are widely used in industrial and medical applications, including pesticide and fertilizer NPs, bio-imaging, drug delivery, and antibacterial (Javed, Ain et al., 2022). The eyes are easily exposed to titanium dioxide NPs. It was found that titanium dioxide NPs induced apoptosis of endothelial cells and increased the proportion of cells in the G2/M phase after primary mouse corneal endothelial cells were exposed to 25 μg/ml for 24 h(Yang, Liu et al., 2021). Combined with the *in vivo* experiments of titanium dioxide NPs, the study showed that the titanium dioxide NPs induced cytotoxicity associated with Nrf2/ARE signaling pathway and its downstream γ-GCS target genes, functional protein ZO-1, and Na-K-ATPase (Yang, Liu et al., 2021). In addition, studies have explored the retinal toxicity of TiO_2_-NPs (Chan, Liao et al., 2021). The vitreous cavity injection of titanium dioxide NPs into C57BL/6 mice disrupted the internal blood-retinal barrier, induced thinned thickness of the inner nuclear layer, increased the thickness of the outer nuclear layer, and decreased the amplitude of the a-wave in the ERG (Chan, Liao et al., 2021).

#### 4.2.6 Zinc oxide nanoparticles

ZnO-NPs have been applied in the medical, cosmetic, and chemical fields, but previous studies have proved their neurotoxicity and immunotoxicity (Keerthana and Kumar 2020). It was shown that 141 nm, 25 μg/ml ZnO-NPs exerted toxic effects on Statens Seruminstitut rabbit keratocytes as evidenced by decreased cell viability, excessive ROS production, increased expression of Bax and TNF-α genes, and decreased expression of Bcl-2 genes (Lee and Park 2019). In a toxicity study on human corneal epithelial cells and conjunctival epithelial cells, 100 nm, 12.5 μg/ml ZnO-NPs produced cytotoxic effects (Li X et al., 2022). They also found that at a concentration of 100 μg/ml, ZnO-NPs would cause excessive production of ROS, induced mitochondrial damage and apoptosis, and inhibited the expression of SIRT1, a factor critical for cell survival in oxidative stress. In addition, other studies found that ZnO-NPs with particle sizes of 10–35 nm disrupted the mitochondrial membrane potential of mouse photoreceptor cells (*661w*), reduced ATP levels, induced the release of cytochrome C, generated excess ROS, enhanced apoptosis, promoted Caspase 3 and Bax expression, and inhibited the expression of Bcl-2 (Wang L et al., 2018).

#### 4.2.7 Silica nanoparticles

Mesoporous silica NPs are promising drug carriers (Sun, Jiang et al., 2019). Our previous study found that the toxicity of 100 μg/ml of 80 nm mesoporous silica NPs on human corneal epithelial cells was presented by apoptosis, oxidative stress, and DNA damage, which were mainly mediated by PI3K-Akt and MAPK signaling pathway (Chen, Zhu et al., 2020). After human corneal epithelial cells were exposed to silica NPs for 48 h, the production of intracellular ROS was increased in a dose-dependent manner (Park, Jeong et al., 2016). In a toxicity assay of silica NPs on trabecular meshwork cells, a slight increase was observed in lactose dehydrogenase at a particle size of 50 nm and a concentration of 100 μg/ml for 48 h. However, the actin skeleton and morphology of the cells remained intact, and no increase in ROS was observed. The same silica NPs injected into rabbit eyes did not present inflammation, edema, acute or chronic toxic effects (Kim, Park et al., 2021).

#### 4.2.8 Carbon nanoparticles

Carbon NPs mainly include carbon nanotubes, fullerenes, graphene, graphene oxide, and reduced graphene oxide. Carbon nanotubes are classified into single-walled carbon nanotubes (SWCNTs) and multi-walled carbon nanotubes (MWCNTs). Kishore et al. found that two different sizes of carbon nanotubes (MWCNT1:5–8 μm in length with an outside diameter of 140 ± 30 nm and inside diameter of 3–8 nm; MWCNT2: 1–10 μm in length with an outside diameter of 10–15 nm and inside diameter of 2–6 nm) damaged rabbit eyes as illustrated by reversible conjunctival reddening and effusion (Kishore et al., 2009). A zero-irritation score was found in the eye irritation assay (Hen Egg Chorion Allantoic Membrane test). Consistently, Ema et al. investigated the irritating effects of two single-walled carbon nanotubes and two multi-walled carbon nanotubes on rabbit eyes (Ema, Matsuda et al., 2011). The results showed that only one type of multi-walled carbon nanotubes caused slight reversible conjunctival congestion, the others were non-irritating to the eyes.

Aoshima et al. conducted experiments on the stimulation of rabbit eyes by high-purity fullerenes (Aoshima, Saitoh et al., 2009). Slight irritation was observed within 24 h, mainly in the form of redness of the conjunctiva and defective corneal epithelium in the rabbit eyes, while these symptoms disappeared after 2 days. The data indicated that irritation was caused by the contact of insoluble high-purity fullerenes with the eye. Ema et al. also performed an acute eye irritation experiment with fullerenes (Ema, Matsuda et al., 2013). The results proved that fullerenes caused conjunctival congestion at 1 h, but not at 24 h. No corneal clouding and iris abnormalities were found after fullerene exposure. Their results suggest that short-term fullerene exposure causes minimal ocular toxicity, but the assessment of long-term fullerene ocular exposure is lacking and further experiments may be needed.

Our previous studies have shown that graphene oxide NP exposure caused toxicity to human primary corneal epithelial cells and human conjunctival epithelial cells in a dose- and time-dependent manner (Wu, Yan et al., 2016). *In vivo* experiments, graphene oxide did not cause toxicity to rabbit eyes, but short-term repeated exposure caused reversible corneal clouding, conjunctival redness, and corneal epithelial damage in Sprague-Dawley rats. Similarly, An et al. conducted *in vivo* and *in vitro* ocular toxicity studies with graphene oxide and reduced graphene oxide (An, Zhang et al., 2018). They found that a high concentration (100 μg/ml) of reduced graphene oxide exposure for 7 days did not cause eye toxicity in mice. In contrast, graphene oxide exposure caused significant intraocular inflammation, corneal cell apoptosis, iris neovascularization production, and apoptosis of corneal epidermal cells in mice.

## 5 Challenge and future

Despite immense progress in the toxicity assessment of NPs, limited studies proved their effects on human health. The safety evaluation of NPs has not been conducted sufficiently.

### 5.1 Chronic nanoparticles toxicology

Human exposure to NPs from natural and anthropogenic sources is inevitable. Therefore, it is necessary to clarify the potential acute or chronic adverse effects of NPs on humans upon constant exposure (Najahi-Missaoui, Arnold et al., 2020). It has been widely accepted that dissolution plays an important role in metallic NP toxicity. Thus, the residence time, transport, and metabolism (biotransformation) of NPs in the body are closely associated with NP toxicity. Animal models with chronic diseases are sensitive to NPs with low levels (Chrishtop, Prilepskii et al., 2021). Assessing the toxicity of NPs in models with chronic diseases could be better to simulate the real environment state. Therefore, the appropriate dosage of NPs needed to be considered in patients suffering from chronic disease.

### 5.2 The human 3D model

Growing evidence has shown the potential adverse effects of NPs from cellular and animal models, while the general questions of nanotoxicology are far from being resolved. Conventional two-dimensional (2D) cell cultures can not recapitulate cell-cell interactions, animal models, and fully reflect human physiological processes due to species differences (Liu, Lin et al., 2020). Recently, with the rapid development of stem cell technology, stem cell-derived human organoids have been used for drug discovery, chemical toxicity, and safety *in vitro* assessment (Li M et al., 2021; Zeng, Li et al., 2021; Li et al., 2022a; Li et al., 2022b). Compared with 2D models, 3D organoid models are more suitable for comprehensively representing *in vivo* physiological microenvironments and cellular interactions (Azhdarzadeh, Saei et al., 2015; Prasad, Kumar et al., 2021). The development of highly adaptable microphysiological systems can provide a more physiological tissue and organ model for nanotoxicological assessment (Ashammakhi, Darabi et al., 2020). Previous 3D microphysiological systems have been developed in the brain, skin, thyroid system, lung, cardiovascular system, liver, kidney, intestine, and spinal cord (Fritsche, Haarmann-Stemmann et al., 2021; Nuciforo and Heim 2021; Lee, Shin et al., 2022). Microphysiological systems combined with engineering strategies could comprehensively monitor the cell responses in real-time under toxic stress and provide a longer lifespan for chronic toxicity studies.

### 5.3 Evaluation of developmental toxicity

Pregnant women are easily exposed to NPs through inhalation, ingestion, and dermal contact. It is well known that NPs could cross the placenta, thus prenatal exposure may cause embryonic developmental toxicity (Aengenheister, Favaro et al., 2021). Therefore, it is necessary to investigate the embryonic developmental toxicity of NPs. However, there are few studies focused on fetal developmental exposure. Although previous studies reported the effects of NPs on the development of zebrafish embryos (Lacave, Retuerto et al., 2016) and mice embryos (Austin, Umbreit et al., 2012; Hong, Kim et al., 2014), it is controversial that these results can be directly applicable to human embryos and fetuses (Dugershaw, Aengenheister et al., 2020; Wang and Wang 2020). Moreover, the indirect developmental toxic effects of NPs may arise from maternal- and placental-derived oxidative stress and inflammation-induced placental impairment and secretion of placental hormones and small molecules (Dugershaw, Aengenheister et al., 2020; Deval, Boland et al., 2021). Recently, the dynamic placenta-on-a-chip model can mimic the real anatomy and physiology of the human placenta, which will enable a new phase of fetal nano-drug toxicology assessment (Aengenheister, Favaro et al., 2021). Evaluating the toxicity of NPs to embryos will help to inform the impact of NPs on maternal-fetal outcomes and help to find a reasonably safe dose that can be used in pregnant women.

## 6 Conclusion

Although NPs have been identified as suitable for ophthalmic drug delivery, a more comprehensive and in-depth NP toxicological assessment is needed to understand the underlying mechanism of the human ocular tissue.
